# Systematic Endobronchial Ultrasound-guided Mediastinal Staging Versus Positron Emission Tomography for Comprehensive Mediastinal Staging in NSCLC Before Radical Radiotherapy of Non-small Cell Lung Cancer: A Pilot Study

**DOI:** 10.1097/MD.0000000000002488

**Published:** 2016-03-03

**Authors:** Daniel P. Steinfort, Shankar Siva, Tracy L. Leong, Morgan Rose, Dishan Herath, Phillip Antippa, David L. Ball, Louis B. Irving

**Affiliations:** From the Department of Cancer Medicine, Peter MacCallum Cancer Institute, East Melbourne (DPS, LBI); Department of Medicine, University of Melbourne (DPS, TLL, LBI); Department of Respiratory Medicine, Royal Melbourne Hospital, Parkville (DPS, MR, LBI); Department of Respiratory Medicine, Monash Medical Centre, Clayton (DPS); Department of Radiation Oncology, Peter MacCallum Cancer Institute, East Melbourne (SS, DLB); Sir Peter MacCallum Department of Oncology, University of Melbourne (SS, DLB); Department of Nuclear Medicine (DG); Department of Cardiothoracic Surgery, Royal Melbourne Hospital, Parkville (PA); and Department of Cancer Surgery, Peter MacCallum Cancer Institute (PA), East Melbourne, Australia.

## Abstract

Despite known limitations of positron emission tomography (PET) for mediastinal staging of non-small cell lung cancer (NSCLC), radiation treatment fields are generally based on PET-identified disease extent. However, no studies have examined the accuracy of FDG-PET/CT on a per-node basis in patients being considered for curative-intent radiotherapy in NSCLC.

In a prospective trial, patients with NSCLC being considered for definitive thoracic radiotherapy (± systemic chemotherapy) underwent minimally invasive systematic mediastinal evaluation with endobronchial ultrasound-guided transbronchial needle aspiration (EBUS-TBNA) following noninvasive staging with integrated PET-CT.

Thirty patients underwent EBUS-TBNA, with TBNA performed from a mean 2.5 lymph node (LN) stations per patient (median 3, range 1–5). Discordant findings between PET-CT and EBUS-TBNA were observed in 10 patients (33%, 95% CI 19%–51%). PET-occult LN metastases were demonstrated by EBUS in 4 patients, whereas a lesser extent of mediastinal involvement, compared with FDG-PET, was demonstrated by EBUS in 6 patients, including 2 patients downstaged from cN3 to pN2. LNs upstaged by EBUS were significantly smaller than nodes downstaged by EBUS, 7.5 mm (range 7–9) versus 12 mm (range 6–21), *P* = 0.005.

A significant proportion of patients considered for definitive radiotherapy (+/-chemotherapy) undergoing systematic mediastinal evaluation with EBUS-TBNA in this study have an extent of mediastinal NSCLC involvement discordant with that indicated by PET-CT. Systematic EBUS-TBNA may aid in defining the extent of mediastinal involvement in NSCLC patients undergoing radiotherapy. Systematic EBUS-TBNA has the potential to contribute significantly to radiotherapy planning and delivery, by either identifying occult nodal metastases, or demonstrating FDG-avid LNs to be disease-free.

## INTRODUCTION

Accurate mediastinal staging of nonsmall cell lung cancer (NSCLC) is critical for determination of optimal treatment strategies. 18F-fluorodeoxyglucose positron emission tomography (FDG-PET) fused with computed tomography (PET-CT) imaging is routinely used for noninvasive staging of patients with suspected or known NSCLC, although mediastinal abnormalities on PET-CT require invasive confirmation due to the limited diagnostic accuracy of PET-CT.^1,2^

Thoracic surgical guidelines identify mediastinal sampling as being selective (involving only selected suspicious nodes), or systematic (exploration and biopsy of a standard set of lymph node [LN] stations in each case).^3,4^ For patients with early-stage (Stage I and II) NSCLC, guidelines recommend systematic intraoperative mediastinal LN sampling or complete mediastinal LN dissection (Grade 1B)^3,5^ to accurately assess the pathologic stage, which is critical to direct adjuvant therapy. Consequently, at completion of therapy, the pathologic extent of disease is discretely defined for surgical patients.

In contrast, although invasive pathologic confirmation of mediastinal NSCLC disease is recommended before radical intent radiotherapy (± systemic chemotherapy), there is no consensus regarding the extent to which pathologic evaluation of the mediastinum should be performed. Thus, although pathological confirmation of mediastinal LN involvement is recommended before radical radiotherapy (with or without chemotherapy),^2^ in contrast to surgical candidates, comprehensive staging of the mediastinum is not routinely performed in this patient group. This is potentially clinically significant, as, despite the imperfect negative- and positive-predictive value of PET,^2,6–10^ radiation treatment fields are generally constructed on the basis of PET-identified disease extent.^11^ Sensitivity of PET/CT is even poorer when individual nodal stations are considered separately.^12^ Hence, any false-positive nodal activity will result in an unnecessarily extensive field of radiation with consequent greater risk of toxicity, whereas PET-occult nodal metastases will result in the risk of geographic miss, increasing the likelihood of local disease recurrence.

Thus, accurate pathologic characterization of the mediastinum in patients receiving radical radiotherapy for NSCLC (±chemotherapy) has the potential to improve treatment outcomes both in terms of disease control and treatment toxicity. One prior study has demonstrated EBUS may detect PET-occult LN metastases in patients being considered for stereotactic radiotherapy for clinical Stage I NSCLC^13^; however, no previous studies have undertaken systematic mediastinal evaluation of patients with locally advanced NSCLC.

We hypothesized that systematic mediastinal evaluation with minimally invasive EBUS-TBNA in NSCLC patients being considered for radical radiation therapy may identify disease extent discrepant of that indicated by PET-CT. This may have significant implications for radiation treatment planning and consequently treatment-related outcomes. We conducted a prospective observational study to examine this hypothesis and findings are presented here.

## METHODS

Melbourne Health Institutional review board approval was granted for performance of this prospective observational study. All patients provided written informed consent.

### Design and Setting

We performed a prospective multicenter observational cohort study in 3 tertiary centers in Melbourne, Australia.

### Patients

Eligible patients were those undergoing mediastinal evaluation with EBUS-TBNA for diagnosis\staging of suspected\known NSCLC wherein noninvasive imaging and\or clinical condition indicated the likely treatment modality would be external beam radiotherapy (±systemic chemotherapy) with curative intent, following discussion at a Lung Cancer Multidisciplinary Meeting. Diagnoses other than NSCLC, and the presence of medical comorbidities precluding bronchoscopy, resulted in exclusion from the cohort.

Patients underwent noninvasive staging with PET-CT before bronchoscopy with pretreatment staging established according to the 7^th^ edition of the Lung Cancer Stage Classification, the TNM descriptors for which are reviewed in detail elsewhere.^14^

### Performance of PET-CT

Integrated PET-CT was performed before EBUS-TBNA in all patients at 3 accredited Australian PET centers according to standard institutional protocols using one of GE discovery 690 (GE Medical Systems, Milwaukee, WI), Discovery STE (GE Medical Systems), or Biograph 64/40 (Siemens Medical Solutions, Malvern, PA).

### Performance of EBUS-TBNA

EBUS-TBNA was performed under conscious sedation as previously described^15,16^ with a dedicated linear array bronchoscope (either BF-UC180F-OL8, Olympus, Tokyo, Japan, or EB-1970UK Pentax, Kashiwa, Japan).

Convex probe EBUS evaluation was performed in a systematic fashion, commencing with the highest contralateral mediastinal (N3) LN, as previously described^17^ LN station anatomy was identified according to endobronchial and sonographic landmarks as previously described.^18^ Any identified LN ≥6 mm in diameter was sampled via EBUS-TBNA, regardless of sonographic findings.^19^

Rapid on-site cytologic examination (ROSE) of TBNA aspirates was performed using a rapid Romanowsky stain (Quick Dip; POCD Scientific, Artarmon, Australia), as previously described.^20^ The aspirate was deemed adequate if lymphocytes were observed, or diagnostic if malignant cells were observed. If inadequate, TBNA was repeated once more. If adequate benign lymphocyte tissue was seen, progression to evaluation of N2 LNs was undertaken, commencing from the most superior PET-negative station, then proceeding as above to inferior N2 stations (eg. 4R\L, 7). Once PET-negative stations were evaluated, we proceeded to EBUS-TBNA of PET-positive LN stations.

For each TBNA, following transfer of initial TBNA material to slides for ROSE, all subsequent materials were placed in formalin solution to allow the preparation of a cell block for histological evaluation and immunohistochemistry (IHC), as previously described.^21^ Pathology outcomes were based on final histocytologic reports following examination of these specimens.

### Review of Discordant Cases

PET-CT imaging for all patients in whom EBUS and PET-CT returned discordant findings regarding the extent of mediastinal involvement underwent independent blinded review to confirm findings from the original PET report. A systematized approach to this was undertaken as follows:

A single PET physician (DG) blinded to the EBUS results and PET-CT reports obtained DICOM image files for the discordant FDG-PET studies and viewed the studies using OsiriX imaging software (Pixmeo, Bermex, Switzerland). Positive nodes were classified as nodes demonstrating FDG uptake greater than mediastinal blood pool uptake. The specific nodal stations assessed were 2R, 2L, 4R, 4L, and 7, and the reviewing PET physician reported each nodal station individually.

The aim of this observational study was to compare diagnostic accuracy of EBUS-TBNA with PET. Where a greater extent of mediastinal involvement was demonstrated by EBUS, this information was incorporated into radiotherapy planning. Despite the high negative predictive value (NPV) of EBUS-TBNA,^22,23,46^ given the surgical risks of mediastinoscopy,^24–26^ wherein EBUS suggested a lesser extent of mediastinal disease than was suggested on PET, planning was undertaken on the basis of PET findings alone.

### Statistical Analysis

Categorical variables were presented as summary statistics, including simple proportions. All reported confidence intervals are 2-sided. Sensitivity, specificity, and accuracy of the 2 methods were calculated according to standard definition. Continuous data were analyzed with the unpaired *t* test with Welch correction using GraphPad InStat (GraphPad Software Inc, La Jolla, CA). For all analyses, the level of statistical significance was set at 0.05.

## RESULTS

**FIGURE 1:**
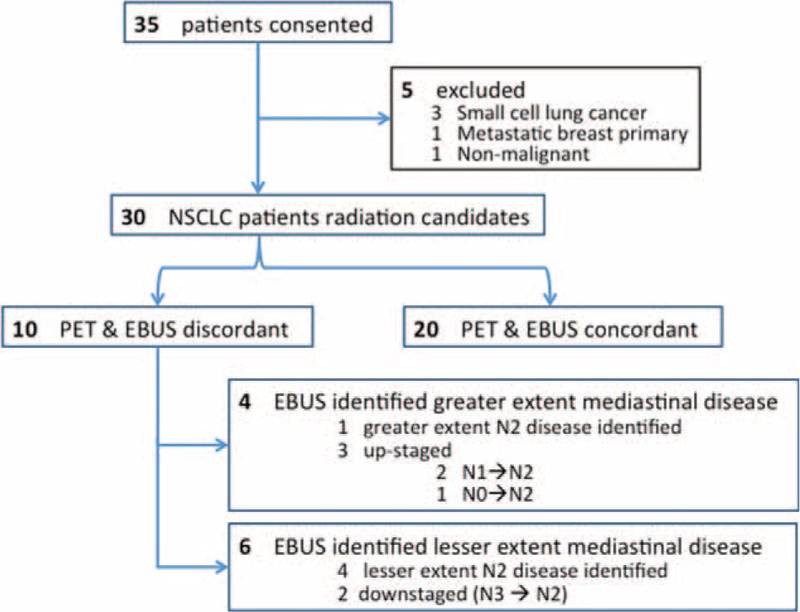
Flowchart of patients enrolled in the study.

Thirty-five patients undergoing EBUS-TBNA consented to inclusion in the study. All patients underwent EBUS within a maximum of 15 days of performance of PET-CT. Five patients were excluded from the study (Figure 1); therefore, 30 eligible patients with NSCLC form the basis of this report. Male:female ratio was 21:9.

No procedural complications occurred during performance of EBUS-TBNA. LNs were visualized by EBUS at a mean 2.9 LN stations per patient. Sampling of visualized LNs was precluded because of size <6 mm (n = 9) or positive ROSE specimen at superior mediastinal LN station (n = 2). Thus, LN sampling was performed from a mean 2.5 LN stations per patient (median 3, range 1–5).

Adequate samples were obtained from all sites examined by EBUS-TBNA. Mean long-axis size of sampled LN was 16 ± 7.8 mm (median 13 mm, range 5–36 mm). Twenty-four percent of sampled LNs were ≤10 mm.

### Comparison of PET and EBUS Findings

Findings regarding the extent of mediastinal disease on PET-CT and EBUS were concordant in 20 of 30 participants (67%, 95% CI 0.49–0.81). T-, and N-stage of these participants are recorded in Table 1.

**TABLE 1:**
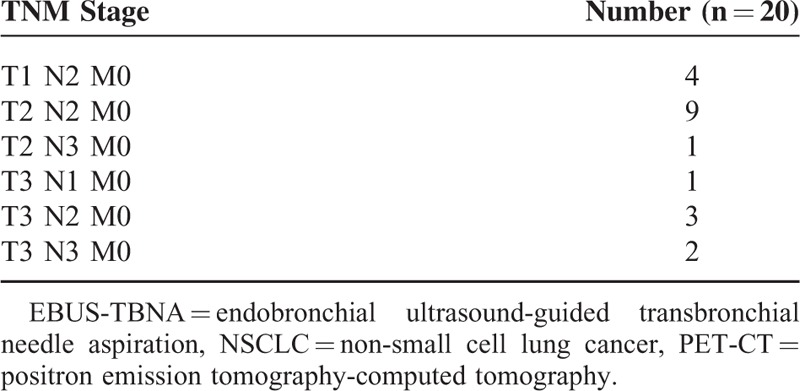
T-, N-, M-stage of patients in whom EBUS-TBNA and PET-CT demonstrated concordant results regarding mediastinal extent of NSCLC involvement

Discordant findings were observed in 10 of 30 patients (33%, 95% CI 0.19–0.51) Detailed information regarding CT-, PET-, and EBUS-identified stage, and findings at the LN stations returning discrepant findings are presented in Table 2.

**TABLE 2:**
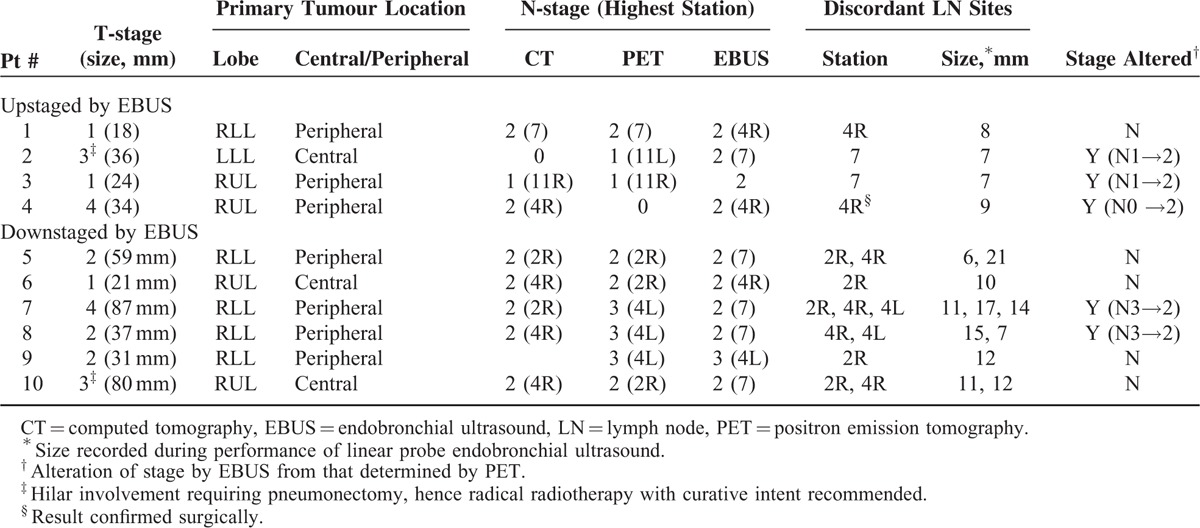
Clinicoradiologic Features of Patients With Discordant Lymph Node Staging Results Between PET and EBUS

EBUS-TBNA identified malignancy in 4 LNs wherein PET-CT had not detected disease, thus identifying a greater extent of mediastinal involvement in 4 patients (false negative on PET). Three patients were upstaged by EBUS-TBNA and in 1 further patient, extent of disease was greater than noted on PET because of more proximal involvement of LN disease not resulting in stage advancement. Median size of LN upstaged by EBUS was 7.5 mm (range 7–9).

EBUS-TBNA demonstrated only benign lymphocytes in 11 mediastinal LNs (6 patients) wherein PET-CT had indicated the presence of disease. Thus, in 6 patients, EBUS identified a lesser extent of mediastinal disease than PET, including 2 patients downstaged from N3 to N2 (50% of all patients staged N3 by PET-CT, 95% CI 15%–85%). Median size of LN downstaged by EBUS was 12 mm (range 6–21).

LNs upstaged were significantly smaller than those downstaged (*P* = 0.005)

## DISCUSSION

Our report is the first to describe discrepancies in mediastinal staging of NSCLC patients between invasive and noninvasive methods in patients with locoregional NSCLC before radical radiotherapy (±systemic chemotherapy). Our results suggest a significant proportion of this group has an extent of mediastinal nodal involvement different to that indicated by noninvasive PET-CT, with clear implications for radiotherapy planning performed on the basis of imaging alone. We identify 33% of such patients have inaccurate characterization of the mediastinum by PET-CT, with EBUS-TBNA demonstrating a greater extent of disease in 4 patients and a lesser extent of disease in 6 (including 2 patients downstaged from N3 to N2.

EBUS-TBNA is a minimally invasive technique with high diagnostic accuracy in mediastinal staging of NSCLC.^16,27^ It has supplanted invasive surgical staging for mediastinal assessment given its equivalent\superior diagnostic performance, and its beneficial safety/morbidity^16,27^ and cost^28^ profiles. It is recommended as the best first test for invasive mediastinal evaluation,^2^ and recent studies confirm sensitivity of EBUS-TBNA is equivalent to, or exceeds, mediastinoscopy.^22,29^ Multiple studies have confirmed the ability of EBUS-TBNA to detect PET-occult LN metastases in clinical stage I NSCLC, and our findings indicate EBUS-TBNA may also identify PET-occult LN metastases within the mediastinum of patients with clinical Stage III NSCLC.

Previous reports suggest a very high NPV of EBUS-TBNA, varying from 0.91 to 0.99.^22,23,46^ In comparison, sensitivity and specificity of PET-CT for detection of mediastinal metastases are estimated at 77% and 86%,^2^ and just 53% and 91%, respectively, when examined on a per-nodal basis.^12^ Consequently, invasive mediastinal staging of FDG-avid lymph nodes is recommended to ensure operable (Stage I and II) patients are not wrongly excluded from potentially curative resection. Such a recommendation regarding FDG-avid nodes in patients in which N2 disease has been confirmed at an alternate site is lacking. Our results indicate that in patients with FDG-avid LN metastases, other sites of FDG-avidity may be false-positive results.

Our observation of variation between EBUS- and PET-determined mediastinal disease extent is consistent with surgical literature, although this variation has not previously been demonstrated in pN2 NSCLC patients undergoing nonsurgical treatment. EBUS detected PET-occult nodal involvement in 13% of our cohort, consistent with the 5% to 16% upstaging rate among patients PET-staged cN0 following surgical^6,7^ or minimally invasive^8–10^ lymph node sampling. Similarly, false-positive PET-CT findings were suggested by EBUS-TBNA in 20% of our cohort, consistent with previously reported specificity of 83% to 87% for PET-CT detection of mediastinal disease wherein prevalence of disease is >20%.^2^

Published reviews have reported a sensitivity of integrated PET-CT of just 62% for detection of mediastinal lymph node metastases.^2^ Importantly, postsurgical studies suggest that the median size of metastatic foci in mediastinal lymph nodes involved with NSCLC is 7 mm.^30^ This is less than the accepted limit of detection of PET/CT^12,31^; therefore, almost certainly a proportion of patients have a mediastinal disease extent greater than that indicated by PET/CT. Such lesions may only be detected by invasive means. Multiple studies have demonstrated the ability of EBUS-TBNA to identify PET-occult N2 disease,^8–10^ with high sensitivity preserved even in evaluation of subcentimeter disease.^10^ Therefore, our finding of EBUS-detected PET-occult disease occurring in nodes with median size 7.5 mm is unsurprising.

Interestingly, 2 of 4 patients upstaged by EBUS were patients with cN1 disease (based on PET-CT). Postoperative pathologic upstaging may occur in over 25% of patients staged cN1,^32^ which likely reflects the increased biologic propensity of tumour to spread to mediastinal nodes given it has already acquired the capacity to metastasize to hilar nodes. Consequently, both ACCP and ESTS guidelines on NSCLC staging recommend (Grade 1C) that invasive staging be performed before resection in those staged cN1 by PET.^2,33^

Accurate characterization of extent of mediastinal disease may have major ramifications for treatment outcomes. In a significant proportion of patients who demonstrate treatment failure following radiotherapy, disease is seen to recur “out-of-field,”^34,35^ thai is, in a location not subjected to radiotherapy. Rates of local disease recurrence are significantly higher in lymph node stations receiving suboptimal radiation doses.^36^ This indicates the importance of accurately defining the extent of disease before radiotherapy for treatment of NSCLC.

The results of this study suggest that systematic mediastinal staging with EBUS may impact the delivery of radiotherapy by either identifying occult nodal metastases (thereby reducing the risk of geographic miss) or demonstrating FDG-avid lymph nodes to be disease-free (thereby allowing reduction in field size and potentially reducing toxicity risks) in a proportion of patients. The dosimetric consequences of discordance of PET/CT- versus EBUS-defined mediastinal staging need to be evaluated in this context. Our findings will be important in informing future studies on systematic mediastinal pathologic staging before radical radiotherapy (±chemotherapy).

Evidence for the potential impact on treatment outcomes for more accurate pathologic staging of the mediastinum may be inferred from studies examining outcomes in patients receiving stereotactic ablative radiotherapy (SABR) for early stage I NSCLC. Higher than expected regional LN failure rates are seen in patients receiving SABR in which staging was performed on the basis of PET alone, with the authors suggesting these outcomes indicate the potential utility of minimally invasive EBUS-TBNA for LN staging before radiation therapy.^37^

We have enrolled a consecutive sample of patients undergoing EBUS-TBNA mediastinal assessment. Previous studies have identified clinical factors (eg, central tumour, cN1, adenocarcinoma histology),^2^ sonographic features,^19^ risk stratification models,^38^ or used artificial neural networks^39^ to identify patients at higher risk of postsurgical upstaging. Larger studies will be required to examine the applicability of such tools to cohorts similar to ours, or to identify specific clinicoradiologic features predictive of a higher rate of detection of occult disease (or false-positive PET findings).

### Limitations

This was a prospective observational pilot study. Surgical confirmation of discrepant results was not performed. Previous meta-analyses have suggested a 0% false-positive rate with EBUS-TBNA,^27^ indicating EBUS-detected disease is a reliable true-positive result. Negative EBUS-TBNA at FDG-avid sites is associated with a NPV of 0.91 to 0.99,^23,40,41,42^ and negative EBUS-TBNA in NSCLC surgical candidates predicts a very low prevalence of metastatic disease in sampled nodes, sufficient to obviate the need for confirmatory mediastinoscopy preoperatively.^40^ Previous studies have confirmed that pathologic staging is the criterion standard for mediastinal staging, with FDG-avidity on PET not associated with risk of recurrence in patients histologically negative mediastinal lymph nodes.^43^ Nevertheless, future studies may consider surgical confirmation of negative EBUS-TBNA results before excluding FDG-avid LN from radiation treatment fields.

We have undertaken systematic staging of the mediastinum. It is unclear whether selective lymph node targeting (eg, the next echelon above LN involved on PET-CT) would identify the same proportion of disease not accurately characterized by PET-CT. “Skip” metastases are a well-recognized phenomena,^44,45^ suggesting that such an approach may reduce accuracy of complete characterization of mediastinal nodes in NSCLC patients before radiation. Potential benefits of selective sampling (eg, reduced procedure time) may be offset by reduced diagnostic performance, although this remains to be examined.

Not all stations were sampled in each patient, with a mean 2.5 LN stations sampled per patient. This is in part because of termination of the procedure following a positive ROSE result, but also is because of no lymph nodes being identified, or only LN <5 mm at a particular station. Although systematic LN sampling was not performed, a systematic EBUS mediastinal examination was performed in each patient.

We have used only EBUS-TBNA in minimally invasive staging. Diagnostic accuracy in mediastinal staging may be improved when both EBUS-TBNA and EUS-FNA are performed,^46–48^ as EUS allows sampling at sites not amenable to EBUS-TBNA (eg. Station 8, 9)^18^; however, this requires an additional procedure. More practically, the linear array EBUS bronchoscope may allow performance of EBUS-TBNA and transoesophageal sampling (EUS-B-FNA) by a single operator. Such an approach may further improve diagnostic accuracy of minimally invasive mediastinal assessment^42,49,50^ and should be considered for future studies.

## CONCLUSIONS

Our pilot study demonstrates that systematic mediastinal staging with EBUS-TBNA in nonsurgical NSCLC patients being considered for radical radiotherapy idenitifes a significant proportion of patients with an extent of mediastinal disease differing from that indicated by non-invasive PET-CT. These results suggest systematic minimally invasive staging should be considered for all patients before definitive thoracic radiotherapy to accurately assess pathologic stage of disease, and to ensure treatment fields most accurately encompass all sites of disease.

Comprehensive mediastinal staging with EBUS may improve the delivery of radiotherapy by either identifying occult nodal metastases (thereby reducing the risk of geographic miss) or demonstrating FDG-avid lymph nodes to be disease-free (thereby allowing reduction in field size and potentially reducing toxicity risks) in a proportion of patients.
